# *The Organon* and Materia Medica Club of the Bay Cities of California

**Published:** 1897-05

**Authors:** W. E. Ledyard

**Affiliations:** Secretary


### *THE ORGANON* AND MATERIA MEDICA CLUB OF THE BAY CITIES OF CALIFORNIA.

The regular semi-monthly meeting was held at the office of Dr. Martin, 606 Sutter Street, San Francisco, on Friday, January 15th, 1897.

The meeting was called to order by the President, Dr. J. M. Selfridge, at 8 P. M.

Present: Drs. J. M. and C. M. Selfridge, Geo. H. Martin, C. J. Holmgren, G. J. Augur, and W. E. Ledyard.

The minutes of the two preceding meetings were read, corrected, and approved.

The motion that the Club henceforth meet in Oakland on the first Friday of the month, and in San Francisco on the third Friday of the month, was carried.

Sections of *The Organon*, 249 to 259, were read and discussed.

Dr. Ledyard, referring to Section 251, has verified the assertion that Bell., Bry., and Rhus must in many instances be repeated before a curative effect takes place.

Again, referring to Section 253, which dwells on the importance of the mental symptoms as a reliable indication of improvement or aggravation, he reported cases of diarrhea, and other morbid discharges, in which the patients said they were no better, although the mental symptoms were greatly relieved.

In such cases one would be apt to make a mistake if not on his guard.

Dr. J. M. Selfridge—In such cases one must not change the remedy, perhaps not even repeat the dose.

Dr. Martin, referring to Section 254, said it was frequently difficult to determine whether the patient is improving, especially in cases in which the mental condition is apt to mislead.

Dr. Augur—In such cases, where patients say they are no better, it is well to do as Hahnemann suggests, take the symptoms seriatim, questioning them with regard to each, and we shall find many symptoms ameliorated.

Dr. Holmgren reported the case of a patient who said he was no better, and "the medicine not worth a nickel," although he was improving steadily. When asked if he could use his hand better, he would say: "No, I can't use it at all." After a while, however, when his attention was attracted to something else, he would use his hand quite freely.

Dr. Augur stated that a patient advised him not to ally himself with a homeopath, notwithstanding he admits that the medicine "acts like a charm."

At one time he had a severe pain, which was greatly aggravated by the slightest movement. Two or three doses of *Bryonia* brought speedy relief, and he was soon asleep. Also, after a cough had been cured, he wanted a bottle of the pellets to carry around with him.

Dr. Martin, discussing Section 257, said: "The habit of prescribing 'favorite medicines' is one that we should continually fight against. I have known so-called homeopathic physicians to prescribe for conditions, *e. g.*, Belladonna for congestion. Nux-vomica for constipation, Aconite for fever, etc."

Dr. J. M. Selfridge—Hahnemann advises against routine practice.

Dr. Ledyard stated that at St. Thomas' Hospital, London, when he was there from 1870 to 1873, several of the physicians had a "hobby" for giving some particular medicine, and invariably incorporated the same in every prescription he made, irrespective of symptoms, idiosyncrasies, or what-not.

Dr. J. M. Selfridge said that Dr. Richard Hughes stated that it was a "hobby" of his to give *Arsenicum* in typhoid fever as soon as diarrhea set in.

Dr. Martin—Hahnemann, in speaking of making provings, says: "A prover must not change his mode of living." In another place he seems to contradict this, by rigidly dieting his patients.

He (Dr. M.) says it doesn't matter how many medicines you give. If one of them is the right medicine, it will act, even in the presence of, and in spite of others.

He mentioned having been in the habit of taking *Silica* for trouble with a filled tooth. For this he once, by mistake, took *Sulph.;* then noticing the mistake he, notwithstanding took *Silica.* Toward morning, for a chill from exposure, took *Aconite*, and both *Silica* and *Aconite* seemed to act.

Dr. Augur—While that may be very true, the probability is we do not get such good results as when we give the single medicine. The most careful prescribers make up their minds most positively before giving the remedy.

Dr. Martin would give the indicated remedy, even if other remedies had recently been given.

Dr. C. M. Selfridge mentioned a case in which *Zinc* had been given. The attending physician waited for the effects of the Zinc to wear off before prescribing, and the patient died.

Dr. J. M. Selfridge reported a case of profuse and long-continued epistaxis cured by a high potency of *Aconite*, after the most powerful styptics had been applied with plugging of posterior nares, and large and repeated doses of ergot without effect. The indication was "fear of death."

Dr. Ledyard reported a case of greatly elongated and swollen uvula, black with the application of Iron and Chlorate of Potash, speedily cured by the highly-potentized medicine.

On motion the Club adjourned to reassemble at the office of Drs. Selfridge, on the first Friday of February, 1897.

W. E. LEDYARD,
*Secretary.*

